# Correction: Štofilová et al. Probiotic-Based Intervention in the Treatment of Ulcerative Colitis: Conventional and New Approaches. *Biomedicines* 2022, *10*, 2236

**DOI:** 10.3390/biomedicines12040797

**Published:** 2024-04-03

**Authors:** Jana Štofilová, Monika Kvaková, Anna Kamlárová, Emília Hijová, Izabela Bertková, Zuzana Guľašová

**Affiliations:** Center of Clinical and Preclinical Research MEDIPARK, Faculty of Medicine, Pavol Jozef Safarik University in Kosice, Trieda SNP 1, 040 11 Kosice, Slovakia; monika.kvakova@upjs.sk (M.K.); anna.kamlarova@upjs.sk (A.K.); emilia.hijova@upjs.sk (E.H.); izabela.bertkova@upjs.sk (I.B.); zuzana.gulasova@upjs.sk (Z.G.)

## Text Correction

The authors would like to add the following clarification regarding the clinical trials evaluating the probiotic product VSL#3 cited in the published paper [1]. A correction has been made to Paragraph 1 in Section 3.2. Effectiveness of Conventional Probiotics in Clinical Trials. “All studies cited in this paper assessed a probiotic formulation previously known as VSL#3. The formulation of the probiotic product VSL#3, which is currently marketed under this brand name, is not identical to the original product, which since 2016 has been known under the generic name ‘De Simone Formulation’ and is available on the market under the brand names Visbiome (USA) and Vivomixx (Europe).”

The authors state that the scientific conclusions are unaffected. This correction was approved by the Academic Editor. The original publication has also been updated.
